# White Adipose Tissue Dysfunction: Pathophysiology and Emergent Measurements

**DOI:** 10.3390/nu15071722

**Published:** 2023-03-31

**Authors:** Natalia Santillana, Camila Astudillo-Guerrero, Amanda D’Espessailles, Gonzalo Cruz

**Affiliations:** 1 Instituto de Nutrición y Tecnología de los Alimentos (INTA), Universidad de Chile, Santiago 8380453, Chile; 2 Centro de Neurobiología y Fisiopatología Integrativa (CENFI), Instituto de Fisiología, Facultad de Ciencias, Universidad de Valparaíso, Valparaíso 2360102, Chile; 3 Programa de Doctorado en Ciencias Mención Neurociencia, Universidad de Valparaíso, Valparaíso 2360102, Chile; 4 Instituto de Ciencias de la Salud, Universidad de O’Higgins, Rancagua 2820000, Chile

**Keywords:** adipose tissue dysfunction, inflammation, obesity, visceral fat, fibrosis

## Abstract

White adipose tissue (AT) dysfunction plays an important role in the development of cardiometabolic alterations associated with obesity. AT dysfunction is characterized by the loss of the expansion capacity of the AT, an increment in adipocyte hypertrophy, and changes in the secretion profile of adipose cells, associated with accumulation of macrophages and inflammation. Since not all people with an excess of adiposity develop comorbidities, it is necessary to find simple tools that can evidence AT dysfunction and allow the detection of those people with the potential to develop metabolic alterations. This review focuses on the current pathophysiological mechanisms of white AT dysfunction and emerging measurements to assess its functionality.

## 1. Introduction

Obesity has become one of the biggest health problems in the world. Many researchers have focused on developing preventive interventions and treatments to combat this problem. However, the long-term impact of those efforts has been modest [1]. In 2015, more than 600 million adults were obese [2]. In this scenario, obesity and the associated chronic low-grade inflammation is now considered one the most important risk factors for developing type 2 diabetes [3,4], cardiovascular dysfunctions [5], and infectious diseases [6], and is currently an important risk factor for COVID-19 hospitalization and death [7].

The study of adipose tissue (AT) has substantially changed in the last decades. Until the late 1940s, AT was considered merely a lipid-containing tissue with no link to metabolism. In the late 1980s and mid-1990s, serum fat-derived factors such as adipsin, tumor-necrosis factor (TNF)-α, and leptin were discovered [8]. In consequence, the original role of AT as an organ that only stores energy was changed by a new concept in which AT is considered an endocrine organ with key roles in energy homeostasis. Since then, studies on the development, function, and pathophysiology of AT have increased substantially. In recent years, the loss of AT functionality has been strongly associated with obesity-induced metabolic alterations [4,9].

Overweight and obesity are defined as abnormal or excessive fat accumulation that presents a risk to health [2]. Under a positive energy balance, AT stores the excess of energy as triglycerides, leading to an expansion of AT. Although this expansion of AT is a physiological mechanism to store energy, an unhealthy expansion of AT is associated with metabolic dysfunctions [10,11]. In this minireview, we review the composition and function of AT and then analyze the pathophysiological mechanisms of AT dysfunction and emerging measurements to assess its functionality in humans.

## 2. Adipose Tissue Composition and Function

Understanding AT structure and function is key to understanding better how it becomes dysfunctional. White AT can typically be organized into two categories: subcutaneous adipose tissue (SAT) and visceral adipose tissue (VAT). SAT includes the gluteus femoral and abdominal or upper-body area, whereas VAT includes both omental and mesenteric depots (also known as intra-abdominal fat) [12,13], but also the fat surrounding the heart [14], and kidneys [15], among others [16].

Within AT, the adipocyte is the cell specializing in the synthesis, storage, and hydrolysis of triglycerides [8]. Adipocytes are surrounded by an extracellular matrix (ECM) and other types of cells such as stem cells, immune cells, endothelial cells, fibroblasts, and preadipocytes, known as the stromal–vascular fraction. Preadipocytes are stem cells that can be recruited to proliferate and differentiate to new adipocytes [17]. Indeed, AT expansion can be the result of either pre-adipocyte proliferation or adipocyte hypertrophy [18]. A healthy AT expansion is characterized by AT enlargement due more to adipocyte proliferation than to adipocyte hypertrophy [19,20], an increased angiogenic response that is proportional to adipose tissue enlargement [21], an adequate extracellular matrix (ECM) remodeling [22], and minimal inflammation.

Adipocytes also secrete specialized molecules called adipokines that have autocrine, paracrine, and endocrine functions [23]. The adipokines include hormones, cytokines, chemokines, growth factors, and the complement system [24]. Intriguingly, the AT also secretes extracellular vesicles that can transport proteins, lipids, and nucleic acids (i.e., microRNAs) that participate in endocrine regulation [25,26].

Each gram of AT contains 1–2 million adipocytes and 4–6 million stromal cells, of which more than half are immune cells [8]. Among the immune cells found in the AT are the macrophages. Macrophages that secrete cytokines such as TNF-α, interleukin (IL)-6, and IL-1β, among others, are described to have an “M1” phenotype, while the anti-inflammatory “M2” macrophages produce immunomodulatory cytokines such as IL-4, IL-10, and IL-13 [27,28]. A healthy expansion of adipose tissue is associated with M2 macrophages instead of M1 ones, which are proinflammatory [29].

The ECM is a network consisting of proteins and proteoglycans that provide structural support and can mediate differentiation, migration, repair, survival, and development of different cells. Importantly, ECM remodeling is required for healthy AT expansion [22]. ECM remodeling is a rearrangement of the ECM due the breakdown of its components by proteases such as metalloproteinases (MMPs). This process permits adipocytes to grow harmonically with adequate ECM replacement and vascularization. During unhealthy AT expansion, ECM remodeling occurs, with an excess in the synthesis of ECM components, leading to fibrosis.

## 3. Adipose Tissue Dysfunction

Fat percentage is highly variable among people, ranging between 5 and 60% of total body weight [30]. In overfeeding, a compensatory increase in total energy expenditure occurs but is usually not enough to equilibrate the intake of energy, leading to AT expansion [31,32,33]. Currently, the AT expansion hypothesis holds that a decreased capacity for SAT expansion favors visceral fat deposition [34,35,36]. This suggests that there is a limit of SAT expandability, which also determinates a genetic susceptibility to develop disorders such as type 2 diabetes [33]. In this sense, we center the discussion on VAT dysfunction.

The term “unhealthy expansion” of AT refers to the expansion of dysfunctional AT, in which there is a hypersecretion of pro-inflammatory adipokines, a decreased secretion of anti-inflammatory adipokines [11,37], a loss of the AT capacity to store energy, and a lack of coordination between adipocyte expansion and extracellular matrix (ECM) remodeling. Unhealthy AT expansion and, consequently, metaflammation leads to an impairment in insulin signaling pathways in the adipocyte, thus decreasing its capacity to store energy [38]. Consequently, a chronic increase in circulating free fatty acids occurs, thus promoting a deposit of lipids in ectopic tissues and, hence, lipotoxicity [39]. Therefore, the current paradigm positions the loss of functionality of AT as a link between obesity and the associated disorders. Remarkably, the AT buffering capacity of the excess of energy is highly variable among individuals [40], and those who that have a low threshold of healthy AT expansion have a phenotype that is metabolically unhealthy, while those who have a high capacity of healthy AT expansion are metabolically healthy, even if they are classified as obese, depending on their BMI.

In adults, fat mass expansion occurs mainly through adipocyte hypertrophy, since just around 8% of adipocytes are renewed each year from preadipocytes [41]. Interestingly, AT expansion in the femoral area is mainly through hyperplasia in adult men and women after 8 weeks in response to overfeeding [12,41,42]. A healthy expansion of AT requires precise coordination between adipocyte hypertrophy/hyperplasia, with adequate vascularization and remodeling of ECM [43,44]. In this sense, when the vasculature does not supply enough irrigation to a zone containing hypertrophic adipocytes, the latter could be exposed to hypoxia.

Healthy AT expansion occurs during an entire lifetime, and studies to clarify the mechanisms by which this expansion occurs are scarce [45]. How excess weight during early life can contribute to the susceptibility to develop unhealthy expansion in adulthood is still unknown. Apparently, there is a genetic susceptibility that determines the threshold to develop unhealthy AT expansion with overfeeding, but this is also influenced by nutritional factors, physical activity, gender, and hormonal status [33]. Omitting the genetic background, all factors preventing inflammation appear to avoid or at least delay unhealthy AT expansion, inflammation being a key marker of AT dysfunction.

### 3.1. Role of ECM Remodeling in Adipose Tissue Dysfunction

Energy availability is variable, and AT needs adequate flexibility to permit its reduction and expansion with changes in energy balance. The flexibility of the ECM permits an adequate adaptation of the adipose tissue to these changes of energy storing, permitting a reorganization of its components (ECM remodeling) when the number and size of adipocytes is modified. Therefore, an altered ECM remodeling during adipose tissue expansion is a feature of AT dysfunction. In this context, an excessive accumulation of ECM components (fibrosis) results from an imbalance between the excessive synthesis of fibrillar components and a slow degradation of these proteins. Chronic overnutrition and AT expansion triggers an excessive synthesis of ECM components due different mechanisms. For example, hypertrophy of adipocytes usually does not allow adequate irrigation of the tissue, thus leading to hypoxia [46,47,48]. Therefore, low oxygen levels lead to molecular adaptations in the cell, such as a high expression of hypoxia inducible factor-1 (HIF-1), which is a transcription factor that increases expression of inflammatory cytokines and ECM components [48,49,50,51]. In addition, the recruitment of inflammatory macrophages (discussed in Section 3.2) leads to an increased inflammatory environment, with several cytokines acting on adipocytes and other cells in the AT [52,53,54]. These cytokines stimulate adipocytes and fibroblasts to produce ECM components [55,56]. The increased expression of ECM proteins, such as collagen, leads to a decreased flexibility of ECM [44]. In fact, collagen VI (an ECM protein) knockout mice consuming a high-fat diet showed larger adipocytes and a better metabolic profile than their wild-type counterparts [57], suggesting that a less rigid ECM would facilitate a functional AT expansion during periods of positive energy balance.

In conclusion, how ECM remodeling occurs is essential as a mechanism leading to AT dysfunction. In this sense, a higher flexibility of the ECM to reorganize its components and adapt to changes in the size and number of adipocytes is a feature of healthy AT expansion while a rigid ECM with an excessive production of its components (e.g., fibrosis) is a feature of unhealthy AT expansion.

### 3.2. Inflammation as a Key Component of AT Dysfunction and Metabolic Impairments

Chronic low-grade inflammation is a key feature of hypertrophied AT in the context of obesity. VAT expansion is associated with an inflammatory environment and immune cell recruitment [26,37]. This local inflammatory environment promotes macrophage infiltration towards the AT, which exacerbates the secretion of proinflammatory cytokines, thus contributing to a systemic inflammatory state [37,53]. In this sense, macrophage infiltration is a key event in the genesis of inflammation and AT dysfunction [58,59,60]. In a proinflammatory context, the M2 macrophages switch to the M1 phenotype, accentuating the imbalance between pro- and anti-inflammatory factors [61]. M1–M2 polarization is a tightly controlled process that responds to environmental changes. Toll-like receptors (TLR) and inflammasomes are key modulators of macrophage polarization. TLR and inflammasomes activate NF-kB and STAT 1 signaling, triggering the inflammatory response in those cells. Wang, Liang and Zen [62], and Castoldi et al. [63] have detailed reviews on the molecular mechanisms subjacent to macrophage polarization and the role of M1 macrophages in metabolic alterations.

In VAT, M1 macrophages infiltrate AT and surround dead hypertrophic adipocytes, forming “crown-like” structures [64,65]. M1 macrophages secrete chemoattractant proteins such as monocyte chemoattractant protein (MCP)-1 (also known as chemokine (C-C motif) ligand 2, CCL2), thus generating a feedforward and exacerbating inflammation [37,66,67]. In addition, immune cell paracrine interaction with adipocytes results in the loss of AT functionality [68] by inhibiting the differentiation of preadipocytes [69,70,71], reducing insulin sensitivity [37,72,73], and decreasing anti-inflammatory adipokine secretion [71,74,75]. The exact molecular pathways initiating macrophage infiltration are unknown. However, adipocyte death and hypoxia can initiate an inflammatory response [76]. Signaling pathways activated by these events are JNK and NF-κB, which control several inflammatory and oxidative cascades [77]. JNK and NF-κB activation increase the production of pro-inflammatory cytokines, endothelial adhesion molecules, and chemotactic proteins, thus promoting monocyte infiltration in VAT and their subsequent differentiation into M1 macrophages [78]. More detailed mechanisms about the relative relevance of local versus infiltrated macrophages in AT dysfunction have previously been described [3,78,79].

Another relevant molecular mechanism involved in AT dysfunction is the activation of the NLRP3 inflammasome. Inflammasomes are protein-signaling platforms that are assembled after the recognition of danger signals. After assembly, pro-caspase 1 is activated, which controls the maturation and secretion of interleukins such as IL-1β and IL-18, which are pro-inflammatory cytokines [80]. Several metabolic stressors such as saturated fatty acids [81], oxidative stress [82], and ceramides [83] activate the inflammasome [84], increasing IL-1β secretion in adipocytes and immune cells. In this context, the expression levels of NLRP3 and IL-1β are increased in the VAT of obese patients with metabolic alterations when compared with obese patients without these alterations [85], suggesting that the NLRP3 inflammasome has an important role in metabolic alterations associated with obesity [86]. In this sense, NLRP3 inflammasome promotes the M1 phenotype in macrophages from the VAT of obese mice [86,87], enhancing the inflammatory status of the AT and contributing to its dysfunction. Interestingly, the blockade of NLRP3 inflammasome activation in human adipocytes decreases the expression of ECM proteins, thus potentially decreasing AT fibrosis [88].

Inflammation also impairs adipogenesis (new adipocyte formation) [89]. In in vitro interaction models, inflammatory factors secreted by macrophages decrease human preadipocyte differentiation into adipocytes but increase proliferation of fibroblasts and promote a profibrotic phenotype [90,91,92]. Thus, an inflammatory environment inhibits adipogenesis, leading to an exacerbated and pathological enlargement of existing adipocytes, thus affecting the healthy expansibility of AT.

In addition to inflammation and ECM remodeling, oxidative stress plays a crucial role in the pathogenesis of metabolic alterations. Oxidative stress is the imbalance between the production of ROS (reactive oxygen species) and antioxidant mechanisms. The mitochondrion is the most important sources of ROS [93]. In this sense, it has been proposed that mitochondrial dysfunction may be a primary cause of AT inflammation [94]. Mitochondria dysfunction is characterized by a decrease in mitochondrial biogenesis, altered membrane potential, a decrease in mitochondrial numbers, and altered activities of oxidative proteins [95]. Recently, Long Xu et al. [96] proposed that mitochondrial dysfunction triggers macrophage polarization, inducing AT inflammation in obesity. These data show the relevance of oxidative stress and mitochondrial function, which have been widely reviewed [95,97,98].

### 3.3. Adipose Tissue Dysfunction and Metabolic Disturbances

The relationship between obesity and insulin resistance (IR) has been widely described, and involves numerous signaling pathways, proteins, adipokines, reactive oxygen species, and inflammation [99]. Insulin signaling disruption is characterized by a decrease in the ability of cells or tissues to respond to physiological levels of insulin [100,101]. The paracrine interaction between M1 macrophage secretion and adipocytes compromises adipose cells’ functionality and promotes insulin signaling disruption [53,102,103]. In this inflammatory context, there is an activation of several proteins, such as c-Jun N-terminal kinase (JNK) and protein kinase C (PKC), as well as transcription factors like nuclear factor (NF)-κB. These signaling pathways are associated with inhibition of insulin-receptor substrates (IRSs) phosphorylation, a decrease in Akt phosphorylation, and an increase in insulin receptor–serine phosphorylation, contributing to the disruption of insulin signaling [104].

Insulin resistance leads to increased lipolysis in AT. Disorders associated with elevated levels of free fatty acids (FFA) in blood and metabolic disturbances of these fatty acids along with intracellular signaling pathways in non-adipose body organs have been termed lipotoxicity [105,106]. Hypertrophic adipocytes release FFA, which activate macrophages, triggering a positive inflammatory loop leading to IR in adipocytes and to fatty acid spillover [39]. In addition, FFA produced by lipolysis in VAT insulin-resistant adipocytes are drained via the portal vein into the liver, contributing to the hepatic deposit of fatty acids [107,108,109]. FFA also are released into the circulation, reaching the pancreas and skeletal muscle, affecting insulin secretion and signaling, and hence glucose uptake [108,109,110,111]. Likewise, the deposition of fatty acids in the heart and kidneys has also been described in an obesity context [109,112]. Thereby, under conditions of chronic overnutrition and loss of healthy AT expandability, surplus energy could be detrimental for the whole organism.

A typical consequence of AT dysfunction is presenting high levels of plasma triglycerides, very low-density lipoprotein (VLDL) and low-density lipoprotein (LDL) [113], and low levels of high-density lipoprotein (HDL) [114,115]. In this sense, the increase in FFA disposal into the liver favors hepatic synthesis of triglycerides and their subsequent increase in plasma. Additionally, hepatic VLDL synthesis is also increased [116]. Furthermore, with the increasing triglycerides in plasma, there is a raised exchange of triglycerides for cholesterol esters in HDL and LDL. Subsequently, the HDL particles become highly enriched in TG and are more susceptible to degradation [117,118,119], leading to low HDL levels in plasma. These low HDL levels are a strong predictor for cardiovascular diseases and mortality [120].

## 4. Current and Emergent Parameters for the Assessment of Adipose Tissue Dysfunction

The impact of obesity on morbidity and mortality is continuously rising in the entire world, and current therapeutic interventions of obesity are initiated too late when people have associated metabolic or cardiovascular disease. In this context, a more accurate diagnosis of obesity, targeting AT dysfunction, would permit classifying people with a high risk for the early development of cardiometabolic disease, independent of their BMI. As AT dysfunction increases, the risk of type 2 diabetes and cardiometabolic diseases increases independently of total fat mass [121,122,123]. The concept of “metabolically healthy obesity” (MHO) has been introduced, defining an obese phenotype in which people have insulin sensitivity, a favorable cardiometabolic profile, and a similar risk of cardiovascular morbidity and mortality compared with individuals of normal weight [124,125,126]. The concept of MHO is still controversial, with no single definition, and there is current discussion about whether this condition is effectively maintained over time [127]. Despite this, the identification of AT functionality could be helpful to classify healthy or unhealthy adipose tissue expansion in clinical practice to prioritize those people susceptible to developing metabolic disorders. Here we present a critical analysis of current and emergent parameters associated with obesity and AT dysfunction. All measurements associated with obesity and adipose dysfunction are summarized in Table 1.

### 4.1. Current Parameters Associated to Obesity

The current classification of obesity, using body mass index (BMI), has several limitations, as BMI is insufficient to account for depot-specific adiposity (as a measure of AT functionality). Moreover, BMI is the quotient of weight and height squared, and is not able to distinguish body composition [134]. In this context, the International Atherosclerosis Society (IAS) and International Chair on Cardiometabolic Risk (ICCR) Working Group on Visceral Obesity recommend that waist circumference should be adopted as a routine measurement in clinical practice, along with BMI, to classify obesity and evaluate cardiovascular risk [135]. Moreover, they propose cut-off points for waist circumference together with BMI to determine risk of future coronary events [130]. Although these recommendations are not a direct measurement of visceral adiposity functionality, it is an interesting and applicable approach to identify an obesity phenotype with higher cardiometabolic risk.

Another indicator widely used in clinical practice to evaluate cardiovascular risk is the diagnosis of metabolic syndrome (MetS). MetS is diagnosed when at least three out of five metabolic alterations are present, following the Alberti criteria [129]: abdominal obesity measured through waist circumference, hypertriglyceridemia, hypertension, low levels of HDL, and high fasting glycemia. The diagnosis of MetS is too late for evaluating the susceptibility to develop metabolic alterations, since the metabolic alterations triggered by adipose dysfunction are already clinically manifested. In this sense, evaluating AT functionality before the development of MetS can be key to the adequate care and treatment of people with adipose dysfunction.

A widely known method for characterizing metabolic risk is through the evaluation of fat deposit distribution through waist circumference. Body fat distribution can be classified either as central adiposity (greater VAT and ectopic fat accumulation associated with cardiometabolic risk) or peripheral adiposity (SAT accumulation in the thighs and hips, less associated with cardiometabolic risk) [130]. Usually, increased fat deposition in SAT is associated with preadipocyte proliferation, a coordination between adipocyte growing and ECM remodeling, adequate vascularization, and insulin sensitivity. Otherwise, VAT is more associated with adipocyte hypertrophy, inflammation, oxidative stress, fibrosis, and insulin resistance. Although waist circumference is the easiest way to measure central adiposity, it does not discriminate between visceral and subcutaneous fat in the abdominal region. Moreover, there is no consensus on the measurement method [135]. More importantly, how do we know if fat accumulation is dysfunctional? Considering that the identification of AT dysfunction cannot be performed solely through one measurement or method (i.e., BMI, or waist circumference), or expensive image analysis (such as DEXA, magnetic resonance imaging (MRI), or computed tomography) several proposals with clinical implications have emerged.

### 4.2. Parameters Associated to AT Dysfunction

#### 4.2.1. Visceral Adiposity Index (VAI)

VAI is a sex-dependent mathematical model that estimates visceral functionality based on BMI, waist circumference, triglycerides, and HDL levels. This index was developed by using data of 1498 healthy normal/overweight subjects and validated through MRI. VAI was inversely related with insulin sensitivity evaluated by a hyperinsulinemic–euglycemic clamp and independently associated with cardiometabolic risk [136]. In a subsequent study, cut-off points were established according to age in the European Caucasian population to identify adipose dysfunction [131]. According to the authors, VAI is a clinical marker of AT dysfunction, able to identify metabolic risk before metabolic syndrome and/or cardiovascular complications develop [137]. Despite VAI being simple to implement, it has some limitations. The applicability of VAI is limited to triglyceride values under 300 mg/dL since hypertriglyceridemia alone may provide more information on the metabolic status, and it may not be useful in the morbid obesity population (BMI > 40 kg/m^2^) [137]. Furthermore, future prospective studies are needed to validate the VAI in non-Caucasian populations.

#### 4.2.2. Hypertriglyceridemic Waist (HTGW)

Not all people with high girth have visceral accumulation, nor are at high risk of type 2 diabetes or cardiovascular disease. HTGW refers to the simultaneous presence of an increased waist circumference and elevated fasting triglyceride concentrations. HTGW represents the loss of the ability to store an energy excess, leading to an increase in both visceral fat depots and blood lipid levels (hypertriglyceridemia) [138]. HTGW has been strongly associated with high VAT content [139,140,141]. The cut-off points proposed are based on gender and have been validated in Canadian [142] and Hispanic populations [143]. HTGW is an accessible tool to estimate AT dysfunction and has been associated with an increased risk of cardiovascular disease and type 2 diabetes in adults [132,144,145] and adolescents [146]. However, more studies that validate cut-off points in different populations and age stratification are still lacking.

#### 4.2.3. Metabolic Flexibility

Metabolic flexibility is defined as the ability of an organism to adapt substrate oxidation to substrate availability [133,147,148]. Metabolic flexibility can be measured through the change in respiratory quotient under metabolic challenges such as overnight fasting or hyperinsulinemic–euglycemic clamp [149,150]. The respiratory quotient (RQ) is defined as the ratio between the volume of CO_2_ produced by the body and the volume of oxygen consumed, and is used as an indicator of substrate use. Thus, values close to 1.0 indicate the use of carbohydrates preferably, while values close to 0.7 and 0.8 indicate the use of triglycerides and proteins, respectively [151,152].

For example, a metabolically flexible subject challenged to a hyperinsulinemic–euglycemic clamp should utilize glucose as a substrate for oxidation, and then the RQ approaches 1. In contrast, a metabolically inflexible subject is unable to adapt substrate oxidation to the substrate availability (in this case glucose), which will be evidenced as a low RQ. Metabolic inflexibility has been proposed as a pathophysiological mechanism for ectopic lipid accumulation and IR [147]. Sparks et al. [153] showed that the presence of small adipocytes from SAT was associated with a higher metabolic flexibility. They also observed an inverse correlation in adipocyte size and metabolic flexibility, suggesting that subjects with larger adipocytes have a less metabolic flexibility than those with smaller adipocytes. Moreover, the presence of large adipocytes in SAT was associated with increased serum inflammatory markers. Noteworthy, the study was carried out in healthy young men, which shows that metabolic alterations can occur in absence of obesity. The contribution of AT to metabolic flexibility disruption, especially the role of VAT, is not yet known. However, it is interesting that, despite how complex the flexibility of metabolism can be, it can also be a potential marker of adipose dysfunction.

#### 4.2.4. Biomarkers

Adiponectin and leptin are widely studied adipokines that could be markers of AT dysfunction and cardiometabolic alterations [154]. Adiponectin is considered a “favorable” adipokine because its actions are related to decreasing inflammation, improving insulin sensitivity, and reducing hepatic gluconeogenesis [155]. In obese individuals, adiponectin levels are decreased and inversely related to pathological states such as diabetes and cardiovascular diseases [156]. Conversely, leptin is a proinflammatory adipokine that induces monocyte proliferation and stimulates proinflammatory cytokine secretion [157]. Leptin could be an important predictor of insulin resistance independent of the degree of obesity [158]. Leptin is also related to the thickness of the carotid intima media, thus predicting atherosclerosis [159]. Since obesity is related to decreased levels of adiponectin and, in turn, to increased levels of leptin, the adiponectin/leptin (A/L) ratio has emerged as a biomarker of AT dysfunction. A decreased A/L ratio is associated with a deleterious secretory profile by the AT [160]. Furthermore, this ratio presents a stronger correlation with insulin resistance than leptin or adiponectin alone [161], which is useful in the identification of those individuals at risk of MetS [162]. Despite the A/L ratio seeming to be a promising indicator, epidemiological studies are needed to establish cut-off points for the different obesity phenotypes.

## 5. Conclusions

Obesity is a pandemic that is still increasing in the world. Cardiometabolic diseases, which are consequences of obesity, are not always dependent on bodyweight and BMI. Instead, they are strongly associated with AT dysfunction. Unfortunately, the histopathological and molecular events occurring during AT dysfunction are difficult to assess. Therefore, more investigation is needed to assess AT dysfunction using proxy measurements that could be important tools contributing to the early identification and classification of patients with high cardiovascular and type 2 diabetes risks. In this context, future epidemiological research is necessary to characterize obesity phenotypes through the measurement and establishment of specific cut-off points for widely studied adipokines, such as leptin or adiponectin, in order to have a more refined diagnosis of those people at risk of developing metabolic abnormalities beyond anthropometric evaluation.

## Figures and Tables

**Table 1:** Measurements associated with obesity and adipose dysfunction.

Measurements Associated to Obesity	Description	Advantages	Disadvantages
BMI [128]	Weight divided by height squared. Depending on its value, classifies the nutritional status of adults as “underweight”, “normal”, “overweight” and “obese”.	Simple and quick to assess in clinical practice.	Does not discriminate body composition or AT dysfunction.
Waist circumference [128,129]	Assesses fat accumulation at the abdominal level.	Simple and quick to assess in clinical practice.	Does not distinguish between VAT and SAT. There is no consensus on the measurement technique.
Metabolic Syndrome [129]	A cluster of risk factors for cardiovascular disease and type 2 diabetes mellitus. Is diagnosed when at least three out of five metabolic alterations are present: abdominal obesity measured through waist circumference, hypertriglyceridemia, hypertension, low levels of HDL, and/or high fasting glycemia.	Well documented in the medical literature.	Diagnosis of metabolic syndrome may be too late to evaluate the susceptibility to develop metabolic alterations.
Waist circumference thresholds within BMI categories [130]	Evaluates risk of future coronary events through waist circumference thresholds stratified by BMI.	Simple and quick to assess in clinical practice. It could improve cardiometabolic risk management in adults with excess adiposity.	It needs to be validated in different populations and ethnic groups.
Measurements associated with adipose dysfunction			
VAI [131]	Mathematical model that estimates visceral functionality based on BMI, waist circumference, triglycerides, and HDL levels.	Inversely related to insulin sensitivity and independently associated with cardiometabolic risk.	It may not be useful in the morbid obesity population (BMI > 40 kg /m^2^). It needs to be validated in non-Caucasian populations.
HTGW [132]	Simultaneous presence of an increased waist circumference and elevated fasting triglyceride concentrations.	Associated with the content of VAT, it could screen those individuals susceptible to developing metabolic alterations more quickly.	It needs to be validated in different populations and ethnic groups.
Metabolic flexibility [133]	Evaluates the ability of an organism to adapt substrate oxidation to substrate availability, measured through the change in respiratory quotient under metabolic challenges such as overnight fasting or hyperinsulinemic–euglycemic clamp.	It could improve the early detection of subjects susceptible to developing metabolic disorders	Studies are needed to validate the impact of VAT dysfunction on metabolic flexibility

BMI: body mass index. VAT: visceral adipose tissue. SAT: subcutaneous adipose tissue. VAI: visceral adiposity index. HTGW: hypertriglyceridemic waist. HDL: high-density lipoprotein.

## Data Availability

Not applicable.

## References

[1] Hafekost K., Lawrence D., Mitrou F., O’Sullivan T.A., Zubrick S.R. (2013). Tackling overweight and obesity: Does the public health message match the science?. BMC Med..

[2] The GBD 2015 Obesity Collaborators (2017). Health Effects of Overweight and Obesity in 195 Countries over 25 Years. N. Engl. J. Med..

[3] Burhans M.S., Hagman D.K., Kuzma J.N., Schmidt K.A., Kratz M. (2018). Contribution of Adipose Tissue Inflammation to the Development of Type 2 Diabetes Mellitus. Compr. Physiol..

[4] Esser N., Legrand-Poels S., Piette J., Scheen A.J., Paquot N. (2014). Inflammation as a link between obesity, metabolic syndrome and type 2 diabetes. Diabetes Res. Clin. Pract..

[5] D’Oria R., Genchi V.A., Caccioppoli C., Calderoni I., Marrano N., Biondi G., Borrelli A., Di Gioia L., Giorgino F., Laviola L. (2022). Impact of Dysfunctional Adipose Tissue Depots on the Cardiovascular System. Int. J. Mol. Sci..

[6] Muscogiuri G., Pugliese G., Laudisio D., Castellucci B., Barrea L., Savastano S., Colao A. (2021). The impact of obesity on immune response to infection: Plausible mechanisms and outcomes. Obes. Rev..

[7] Stefan N., Birkenfeld A.L., Schulze M.B., Ludwig D.S. (2020). Obesity and impaired metabolic health in patients with COVID-19. Nat. Rev. Endocrinol..

[8] Rosen E.D., Spiegelman B.M. (2014). What we talk about when we talk about fat. Cell.

[9] Engin A.B. (2017). Adipocyte-Macrophage Cross-Talk in Obesity. Adv. Exp. Med. Biol..

[10] Bluher M. (2013). Adipose tissue dysfunction contributes to obesity related metabolic diseases. Best Pract. Res. Clin. Endocrinol. Metab..

[11] Longo M., Zatterale F., Naderi J., Parrillo L., Formisano P., Raciti G.A., Beguinot F., Miele C. (2019). Adipose Tissue Dysfunction as Determinant of Obesity-Associated Metabolic Complications. Int. J. Mol. Sci..

[12] Tchkonia T., Thomou T., Zhu Y., Karagiannides I., Pothoulakis C., Jensen M.D., Kirkland J.L. (2013). Mechanisms and metabolic implications of regional differences among fat depots. Cell Metab..

[13] Arner P., Ryden M. (2022). Human white adipose tissue: A highly dynamic metabolic organ. J. Intern. Med..

[14] Gaborit B., Sengenes C., Ancel P., Jacquier A., Dutour A. (2017). Role of Epicardial Adipose Tissue in Health and Disease: A Matter of Fat?. Compr. Physiol..

[15] Grigoras A., Balan R.A., Caruntu I.D., Giusca S.E., Lozneanu L., Avadanei R.E., Rusu A., Riscanu L.A., Amalinei C. (2021). Perirenal Adipose Tissue-Current Knowledge and Future Opportunities. J. Clin. Med..

[16] Schafer K., Drosos I., Konstantinides S. (2017). Perivascular adipose tissue: Epiphenomenon or local risk factor?. Int. J. Obes..

[17] Berry R., Rodeheffer M.S. (2013). Characterization of the adipocyte cellular lineage in vivo. Nat. Cell Biol..

[18] Wang Q.A., Tao C., Gupta R.K., Scherer P.E. (2013). Tracking adipogenesis during white adipose tissue development, expansion and regeneration. Nat. Med..

[19] McLaughlin T., Craig C., Liu L.F., Perelman D., Allister C., Spielman D., Cushman S.W. (2016). Adipose Cell Size and Regional Fat Deposition as Predictors of Metabolic Response to Overfeeding in Insulin-Resistant and Insulin-Sensitive Humans. Diabetes.

[20] Kim S.M., Lun M., Wang M., Senyo S.E., Guillermier C., Patwari P., Steinhauser M.L. (2014). Loss of white adipose hyperplastic potential is associated with enhanced susceptibility to insulin resistance. Cell Metab..

[21] Cao Y. (2007). Angiogenesis modulates adipogenesis and obesity. J. Clin. Investig..

[22] Ruiz-Ojeda F.J., Mendez-Gutierrez A., Aguilera C.M., Plaza-Diaz J. (2019). Extracellular Matrix Remodeling of Adipose Tissue in Obesity and Metabolic Diseases. Int. J. Mol. Sci..

[23] Fasshauer M., Bluher M. (2015). Adipokines in health and disease. Trends Pharmacol. Sci..

[24] Kirichenko T.V., Markina Y.V., Bogatyreva A.I., Tolstik T.V., Varaeva Y.R., Starodubova A.V. (2022). The Role of Adipokines in Inflammatory Mechanisms of Obesity. Int. J. Mol. Sci..

[25] Arner E., Mejhert N., Kulyte A., Balwierz P.J., Pachkov M., Cormont M., Lorente-Cebrian S., Ehrlund A., Laurencikiene J., Heden P. (2012). Adipose tissue microRNAs as regulators of CCL2 production in human obesity. Diabetes.

[26] Kappeler L. (2022). Role of Adipose Tissue microRNAs in the Onset of Metabolic Diseases and Implications in the Context of the DOHaD. Cells.

[27] Yao J., Wu D., Qiu Y. (2022). Adipose tissue macrophage in obesity-associated metabolic diseases. Front. Immunol..

[28] Li C., Xu M.M., Wang K., Adler A.J., Vella A.T., Zhou B. (2018). Macrophage polarization and meta-inflammation. Transl. Res..

[29] Ruggiero A.D., Key C.C., Kavanagh K. (2021). Adipose Tissue Macrophage Polarization in Healthy and Unhealthy Obesity. Front. Nutr..

[30] Lee M.J., Wu Y., Fried S.K. (2013). Adipose tissue heterogeneity: Implication of depot differences in adipose tissue for obesity complications. Mol. Asp. Med..

[31] Leibel R.L., Rosenbaum M., Hirsch J. (1995). Changes in energy expenditure resulting from altered body weight. N. Engl. J. Med..

[32] Schlogl M., Piaggi P., Pannacciuli N., Bonfiglio S.M., Krakoff J., Thearle M.S. (2015). Energy Expenditure Responses to Fasting and Overfeeding Identify Phenotypes Associated with Weight Change. Diabetes.

[33] Cuthbertson D.J., Steele T., Wilding J.P., Halford J.C., Harrold J.A., Hamer M., Karpe F. (2017). What have human experimental overfeeding studies taught us about adipose tissue expansion and susceptibility to obesity and metabolic complications?. Int. J. Obes..

[34] Iacobini C., Pugliese G., Blasetti Fantauzzi C., Federici M., Menini S. (2019). Metabolically healthy versus metabolically unhealthy obesity. Metabolism.

[35] Ryden M., Andersson D.P., Bergstrom I.B., Arner P. (2014). Adipose tissue and metabolic alterations: Regional differences in fat cell size and number matter, but differently: A cross-sectional study. J. Clin. Endocrinol. Metab..

[36] White U., Beyl R.A., Ravussin E. (2022). A higher proportion of small adipocytes is associated with increased visceral and ectopic lipid accumulation during weight gain in response to overfeeding in men. Int. J. Obes..

[37] Kawai T., Autieri M.V., Scalia R. (2021). Adipose tissue inflammation and metabolic dysfunction in obesity. Am. J. Physiol.-Cell Physiol..

[38] Hotamisligil G.S. (2017). Inflammation, metaflammation and immunometabolic disorders. Nature.

[39] Carobbio S., Pellegrinelli V., Vidal-Puig A. (2017). Adipose Tissue Function and Expandability as Determinants of Lipotoxicity and the Metabolic Syndrome. Adv. Exp. Med. Biol..

[40] Taylor R., Holman R.R. (2015). Normal weight individuals who develop type 2 diabetes: The personal fat threshold. Clin. Sci..

[41] Johannsen D.L., Tchoukalova Y., Tam C.S., Covington J.D., Xie W., Schwarz J.M., Bajpeyi S., Ravussin E. (2014). Effect of 8 weeks of overfeeding on ectopic fat deposition and insulin sensitivity: Testing the “adipose tissue expandability” hypothesis. Diabetes Care.

[42] Tchoukalova Y.D., Votruba S.B., Tchkonia T., Giorgadze N., Kirkland J.L., Jensen M.D. (2010). Regional differences in cellular mechanisms of adipose tissue gain with overfeeding. Proc. Natl. Acad. Sci. USA.

[43] Corvera S., Solivan-Rivera J., Yang Loureiro Z. (2022). Angiogenesis in adipose tissue and obesity. Angiogenesis.

[44] Crewe C., An Y.A., Scherer P.E. (2017). The ominous triad of adipose tissue dysfunction: Inflammation, fibrosis and impaired angiogenesis. J. Clin. Investig..

[45] Orsso C.E., Colin-Ramirez E., Field C.J., Madsen K.L., Prado C.M., Haqq A.M. (2020). Adipose Tissue Development and Expansion from the Womb to Adolescence: An Overview. Nutrients.

[46] Blaak E.E., van Baak M.A., Kemerink G.J., Pakbiers M.T., Heidendal G.A., Saris W.H. (1995). Beta-adrenergic stimulation and abdominal subcutaneous fat blood flow in lean, obese, and reduced-obese subjects. Metabolism.

[47] Goossens G.H., Bizzarri A., Venteclef N., Essers Y., Cleutjens J.P., Konings E., Jocken J.W., Cajlakovic M., Ribitsch V., Clement K. (2011). Increased adipose tissue oxygen tension in obese compared with lean men is accompanied by insulin resistance, impaired adipose tissue capillarization and inflammation. Circulation.

[48] Trayhurn P. (2013). Hypoxia and adipose tissue function and dysfunction in obesity. Physiol. Rev..

[49] Fujisaka S., Usui I., Ikutani M., Aminuddin A., Takikawa A., Tsuneyama K., Mahmood A., Goda N., Nagai Y., Takatsu K. (2013). Adipose tissue hypoxia induces inflammatory M1 polarity of macrophages in an HIF-1alpha-dependent and HIF-1alpha-independent manner in obese mice. Diabetologia.

[50] Babapoor-Farrokhran S., Gill D., Alzubi J., Mainigi S.K. (2021). Atrial fibrillation: The role of hypoxia-inducible factor-1-regulated cytokines. Mol. Cell. Biochem..

[51] Darby I.A., Hewitson T.D. (2016). Hypoxia in tissue repair and fibrosis. Cell Tissue Res..

[52] Weisberg S.P., McCann D., Desai M., Rosenbaum M., Leibel R.L., Ferrante A.W. (2003). Obesity is associated with macrophage accumulation in adipose tissue. J. Clin. Investig..

[53] Lee B.C., Lee J. (2014). Cellular and molecular players in adipose tissue inflammation in the development of obesity-induced insulin resistance. Biochim. Biophys. Acta.

[54] Rausch M.E., Weisberg S., Vardhana P., Tortoriello D.V. (2008). Obesity in C57BL/6J mice is characterized by adipose tissue hypoxia and cytotoxic T-cell infiltration. Int. J. Obes..

[55] Henegar C., Tordjman J., Achard V., Lacasa D., Cremer I., Guerre-Millo M., Poitou C., Basdevant A., Stich V., Viguerie N. (2008). Adipose tissue transcriptomic signature highlights the pathological relevance of extracellular matrix in human obesity. Genome Biol..

[56] Johnston E.K., Abbott R.D. (2023). Adipose Tissue Paracrine-, Autocrine-, and Matrix-Dependent Signaling during the Development and Progression of Obesity. Cells.

[57] Khan T., Muise E.S., Iyengar P., Wang Z.V., Chandalia M., Abate N., Zhang B.B., Bonaldo P., Chua S., Scherer P.E. (2009). Metabolic dysregulation and adipose tissue fibrosis: Role of collagen VI. Mol. Cell. Biol..

[58] Martinez-Santibanez G., Lumeng C.N. (2014). Macrophages and the regulation of adipose tissue remodeling. Annu. Rev. Nutr..

[59] Boutens L., Stienstra R. (2016). Adipose tissue macrophages: Going off track during obesity. Diabetologia.

[60] Liang W., Qi Y., Yi H., Mao C., Meng Q., Wang H., Zheng C. (2022). The Roles of Adipose Tissue Macrophages in Human Disease. Front. Immunol..

[61] Hammarstedt A., Gogg S., Hedjazifar S., Nerstedt A., Smith U. (2018). Impaired Adipogenesis and Dysfunctional Adipose Tissue in Human Hypertrophic Obesity. Physiol. Rev..

[62] Wang N., Liang H., Zen K. (2014). Molecular mechanisms that influence the macrophage m1-m2 polarization balance. Front. Immunol..

[63] Castoldi A., Naffah de Souza C., Camara N.O., Moraes-Vieira P.M. (2015). The Macrophage Switch in Obesity Development. Front. Immunol..

[64] Cinti S., Mitchell G., Barbatelli G., Murano I., Ceresi E., Faloia E., Wang S., Fortier M., Greenberg A.S., Obin M.S. (2005). Adipocyte death defines macrophage localization and function in adipose tissue of obese mice and humans. J. Lipid Res..

[65] Murano I., Barbatelli G., Parisani V., Latini C., Muzzonigro G., Castellucci M., Cinti S. (2008). Dead adipocytes, detected as crown-like structures, are prevalent in visceral fat depots of genetically obese mice. J. Lipid Res..

[66] Kanda H., Tateya S., Tamori Y., Kotani K., Hiasa K., Kitazawa R., Kitazawa S., Miyachi H., Maeda S., Egashira K. (2006). MCP-1 contributes to macrophage infiltration into adipose tissue, insulin resistance, and hepatic steatosis in obesity. J. Clin. Investig..

[67] Weisberg S.P., Hunter D., Huber R., Lemieux J., Slaymaker S., Vaddi K., Charo I., Leibel R.L., Ferrante A.W. (2006). CCR2 modulates inflammatory and metabolic effects of high-fat feeding. J. Clin. Investig..

[68] Lu J., Zhao J., Meng H., Zhang X. (2019). Adipose Tissue-Resident Immune Cells in Obesity and Type 2 Diabetes. Front. Immunol..

[69] Keophiphath M., Achard V., Henegar C., Rouault C., Clement K., Lacasa D. (2009). Macrophage-secreted factors promote a profibrotic phenotype in human preadipocytes. Mol. Endocrinol..

[70] Sorisky A., Molgat A.S., Gagnon A. (2013). Macrophage-induced adipose tissue dysfunction and the preadipocyte: Should I stay (and differentiate) or should I go?. Adv. Nutr..

[71] Liu F., He J., Wang H., Zhu D., Bi Y. (2020). Adipose Morphology: A Critical Factor in Regulation of Human Metabolic Diseases and Adipose Tissue Dysfunction. Obes. Surg..

[72] Chung S., Lapoint K., Martinez K., Kennedy A., Boysen Sandberg M., McIntosh M.K. (2006). Preadipocytes mediate lipopolysaccharide-induced inflammation and insulin resistance in primary cultures of newly differentiated human adipocytes. Endocrinology.

[73] Lee Y.S., Li P., Huh J.Y., Hwang I.J., Lu M., Kim J.I., Ham M., Talukdar S., Chen A., Lu W.J. (2011). Inflammation is necessary for long-term but not short-term high-fat diet-induced insulin resistance. Diabetes.

[74] Kern P.A., Di Gregorio G.B., Lu T., Rassouli N., Ranganathan G. (2003). Adiponectin expression from human adipose tissue: Relation to obesity, insulin resistance, and tumor necrosis factor-alpha expression. Diabetes.

[75] Gariballa S., Alkaabi J., Yasin J., Al Essa A. (2019). Total adiponectin in overweight and obese subjects and its response to visceral fat loss. BMC Endocr. Disord..

[76] Reilly S.M., Saltiel A.R. (2017). Adapting to obesity with adipose tissue inflammation. Nat. Rev. Endocrinol..

[77] Hernandez E.D., Lee S.J., Kim J.Y., Duran A., Linares J.F., Yajima T., Muller T.D., Tschop M.H., Smith S.R., Diaz-Meco M.T. (2014). A macrophage NBR1-MEKK3 complex triggers JNK-mediated adipose tissue inflammation in obesity. Cell Metab..

[78] Zatterale F., Longo M., Naderi J., Raciti G.A., Desiderio A., Miele C., Beguinot F. (2019). Chronic Adipose Tissue Inflammation Linking Obesity to Insulin Resistance and Type 2 Diabetes. Front. Physiol..

[79] Bai Y., Sun Q. (2015). Macrophage recruitment in obese adipose tissue. Obes. Rev..

[80] Broz P., Dixit V.M. (2016). Inflammasomes: Mechanism of assembly, regulation and signalling. Nat. Rev. Immunol..

[81] Reynolds C.M., McGillicuddy F.C., Harford K.A., Finucane O.M., Mills K.H., Roche H.M. (2012). Dietary saturated fatty acids prime the NLRP3 inflammasome via TLR4 in dendritic cells-implications for diet-induced insulin resistance. Mol. Nutr. Food Res..

[82] Sharma A., Tate M., Mathew G., Vince J.E., Ritchie R.H., de Haan J.B. (2018). Oxidative Stress and NLRP3-Inflammasome Activity as Significant Drivers of Diabetic Cardiovascular Complications: Therapeutic Implications. Front. Physiol..

[83] Legrand-Poels S., Esser N., L’Homme L., Scheen A., Paquot N., Piette J. (2014). Free fatty acids as modulators of the NLRP3 inflammasome in obesity/type 2 diabetes. Biochem. Pharmacol..

[84] Kelley N., Jeltema D., Duan Y., He Y. (2019). The NLRP3 Inflammasome: An Overview of Mechanisms of Activation and Regulation. Int. J. Mol. Sci..

[85] Esser N., L’Homme L., De Roover A., Kohnen L., Scheen A.J., Moutschen M., Piette J., Legrand-Poels S., Paquot N. (2013). Obesity phenotype is related to NLRP3 inflammasome activity and immunological profile of visceral adipose tissue. Diabetologia.

[86] Wu K.K., Cheung S.W., Cheng K.K. (2020). NLRP3 Inflammasome Activation in Adipose Tissues and Its Implications on Metabolic Diseases. Int. J. Mol. Sci..

[87] Vandanmagsar B., Youm Y.H., Ravussin A., Galgani J.E., Stadler K., Mynatt R.L., Ravussin E., Stephens J.M., Dixit V.D. (2011). The NLRP3 inflammasome instigates obesity-induced inflammation and insulin resistance. Nat. Med..

[88] Unamuno X., Gomez-Ambrosi J., Ramirez B., Rodriguez A., Becerril S., Valenti V., Moncada R., Silva C., Salvador J., Fruhbeck G. (2021). NLRP3 inflammasome blockade reduces adipose tissue inflammation and extracellular matrix remodeling. Cell. Mol. Immunol..

[89] Jiang N., Li Y., Shu T., Wang J. (2019). Cytokines and inflammation in adipogenesis: An updated review. Front. Med..

[90] Bourlier V., Sengenes C., Zakaroff-Girard A., Decaunes P., Wdziekonski B., Galitzky J., Villageois P., Esteve D., Chiotasso P., Dani C. (2012). TGFbeta family members are key mediators in the induction of myofibroblast phenotype of human adipose tissue progenitor cells by macrophages. PLoS ONE.

[91] Tanaka M., Ikeda K., Suganami T., Komiya C., Ochi K., Shirakawa I., Hamaguchi M., Nishimura S., Manabe I., Matsuda T. (2014). Macrophage-inducible C-type lectin underlies obesity-induced adipose tissue fibrosis. Nat. Commun..

[92] Rabhi N., Desevin K., Belkina A.C., Tilston-Lunel A., Varelas X., Layne M.D., Farmer S.R. (2022). Obesity-induced senescent macrophages activate a fibrotic transcriptional program in adipocyte progenitors. Life Sci. Alliance.

[93] Murphy M.P. (2009). How mitochondria produce reactive oxygen species. Biochem. J..

[94] Woo C.Y., Jang J.E., Lee S.E., Koh E.H., Lee K.U. (2019). Mitochondrial Dysfunction in Adipocytes as a Primary Cause of Adipose Tissue Inflammation. Diabetes Metab. J..

[95] Ruiz-Ojeda F.J., Ozla J., Gil Á., Concepción M.A., del Moral A.M., María C. (2018). Chapter 1—Oxidative Stress and Inflammation in Obesity and Metabolic Syndrome. Obesity.

[96] Xu L., Yan X., Zhao Y., Wang J., Liu B., Yu S., Fu J., Liu Y., Su J. (2022). Macrophage Polarization Mediated by Mitochondrial Dysfunction Induces Adipose Tissue Inflammation in Obesity. Int. J. Mol. Sci..

[97] Masschelin P.M., Cox A.R., Chernis N., Hartig S.M. (2019). The Impact of Oxidative Stress on Adipose Tissue Energy Balance. Front. Physiol..

[98] Manna P., Jain S.K. (2015). Obesity, Oxidative Stress, Adipose Tissue Dysfunction and the Associated Health Risks: Causes and Therapeutic Strategies. Metab. Syndr. Relat. Disord..

[99] Petersen M.C., Shulman G.I. (2018). Mechanisms of Insulin Action and Insulin Resistance. Physiol. Rev..

[100] Lee S.H., Park S.Y., Choi C.S. (2022). Insulin Resistance: From Mechanisms to Therapeutic Strategies. Diabetes Metab. J..

[101] Khalid M., Alkaabi J., Khan M.A.B., Adem A. (2021). Insulin Signal Transduction Perturbations in Insulin Resistance. Int. J. Mol. Sci..

[102] Glass C.K., Olefsky J.M. (2012). Inflammation and lipid signaling in the etiology of insulin resistance. Cell Metab..

[103] Lauterbach M.A., Wunderlich F.T. (2017). Macrophage function in obesity-induced inflammation and insulin resistance. Pflügers Arch.-Eur. J. Physiol..

[104] Boucher J., Kleinridders A., Kahn C.R. (2014). Insulin receptor signaling in normal and insulin-resistant states. Cold Spring Harb. Perspect. Biol..

[105] Yazici D., Sezer H. (2017). Insulin Resistance, Obesity and Lipotoxicity. Adv. Exp. Med. Biol..

[106] Engin A.B. (2017). What Is Lipotoxicity?. Adv. Exp. Med. Biol..

[107] Nielsen S., Guo Z., Johnson C.M., Hensrud D.D., Jensen M.D. (2004). Splanchnic lipolysis in human obesity. J. Clin. Investig..

[108] Sears B., Perry M. (2015). The role of fatty acids in insulin resistance. Lipids Health Dis..

[109] Sobczak A.I.S., Blindauer C.A., Stewart A.J. (2019). Changes in Plasma Free Fatty Acids Associated with Type-2 Diabetes. Nutrients.

[110] Oh Y.S., Bae G.D., Baek D.J., Park E.Y., Jun H.S. (2018). Fatty Acid-Induced Lipotoxicity in Pancreatic Beta-Cells During Development of Type 2 Diabetes. Front. Endocrinol..

[111] Tumova J., Andel M., Trnka J. (2016). Excess of free fatty acids as a cause of metabolic dysfunction in skeletal muscle. Physiol. Res..

[112] Nishi H., Higashihara T., Inagi R. (2019). Lipotoxicity in Kidney, Heart, and Skeletal Muscle Dysfunction. Nutrients.

[113] Lessard J., Laforest S., Pelletier M., Leboeuf M., Blackburn L., Tchernof A. (2014). Low abdominal subcutaneous preadipocyte adipogenesis is associated with visceral obesity, visceral adipocyte hypertrophy, and a dysmetabolic state. Adipocyte.

[114] Veilleux A., Caron-Jobin M., Noel S., Laberge P.Y., Tchernof A. (2011). Visceral adipocyte hypertrophy is associated with dyslipidemia independent of body composition and fat distribution in women. Diabetes.

[115] Su X., Peng D. (2020). The exchangeable apolipoproteins in lipid metabolism and obesity. Clin. Chim. Acta.

[116] Jung U.J., Choi M.S. (2014). Obesity and its metabolic complications: The role of adipokines and the relationship between obesity, inflammation, insulin resistance, dyslipidemia and nonalcoholic fatty liver disease. Int. J. Mol. Sci..

[117] Rashid S., Barrett P.H., Uffelman K.D., Watanabe T., Adeli K., Lewis G.F. (2002). Lipolytically modified triglyceride-enriched HDLs are rapidly cleared from the circulation. Arterioscler. Thromb. Vasc. Biol..

[118] Rashid S., Trinh D.K., Uffelman K.D., Cohn J.S., Rader D.J., Lewis G.F. (2003). Expression of human hepatic lipase in the rabbit model preferentially enhances the clearance of triglyceride-enriched versus native high-density lipoprotein apolipoprotein A-I. Circulation.

[119] Stadler J.T., Marsche G. (2020). Obesity-Related Changes in High-Density Lipoprotein Metabolism and Function. Int. J. Mol. Sci..

[120] Zhong G.C., Huang S.Q., Peng Y., Wan L., Wu Y.Q., Hu T.Y., Hu J.J., Hao F.B. (2020). HDL-C is associated with mortality from all causes, cardiovascular disease and cancer in a J-shaped dose-response fashion: A pooled analysis of 37 prospective cohort studies. Eur. J. Prev. Cardiol..

[121] Neeland I.J., Turer A.T., Ayers C.R., Powell-Wiley T.M., Vega G.L., Farzaneh-Far R., Grundy S.M., Khera A., McGuire D.K., de Lemos J.A. (2012). Dysfunctional adiposity and the risk of prediabetes and type 2 diabetes in obese adults. JAMA.

[122] Jung S.H., Ha K.H., Kim D.J. (2016). Visceral Fat Mass Has Stronger Associations with Diabetes and Prediabetes than Other Anthropometric Obesity Indicators among Korean Adults. Yonsei Med. J..

[123] Piche M.E., Tchernof A., Despres J.P. (2020). Obesity Phenotypes, Diabetes, and Cardiovascular Diseases. Circ. Res..

[124] Wildman R.P., Muntner P., Reynolds K., McGinn A.P., Rajpathak S., Wylie-Rosett J., Sowers M.R. (2008). The obese without cardiometabolic risk factor clustering and the normal weight with cardiometabolic risk factor clustering: Prevalence and correlates of 2 phenotypes among the US population (NHANES 1999–2004). Arch. Intern. Med..

[125] Wang B., Zhuang R., Luo X., Yin L., Pang C., Feng T., You H., Zhai Y., Ren Y., Zhang L. (2015). Prevalence of Metabolically Healthy Obese and Metabolically Obese but Normal Weight in Adults Worldwide: A Meta-Analysis. Horm. Metab. Res..

[126] Bluher M. (2020). Metabolically Healthy Obesity. Endocr. Rev..

[127] Tsatsoulis A., Paschou S.A. (2020). Metabolically Healthy Obesity: Criteria, Epidemiology, Controversies, and Consequences. Curr. Obes. Rep..

[128] (1998). Executive summary of the clinical guidelines on the identification, evaluation, and treatment of overweight and obesity in adults. Arch. Intern. Med..

[129] Alberti K.G., Eckel R.H., Grundy S.M., Zimmet P.Z., Cleeman J.I., Donato K.A., Fruchart J.C., James W.P., Loria C.M., Smith S.C. (2009). Harmonizing the metabolic syndrome: A joint interim statement of the International Diabetes Federation Task Force on Epidemiology and Prevention; National Heart, Lung, and Blood Institute; American Heart Association; World Heart Federation; International Atherosclerosis Society; and International Association for the Study of Obesity. Circulation.

[130] Ross R., Neeland I.J., Yamashita S., Shai I., Seidell J., Magni P., Santos R.D., Arsenault B., Cuevas A., Hu F.B. (2020). Waist circumference as a vital sign in clinical practice: A Consensus Statement from the IAS and ICCR Working Group on Visceral Obesity. Nat. Rev. Endocrinol..

[131] Amato M.C., Giordano C., Pitrone M., Galluzzo A. (2011). Cut-off points of the visceral adiposity index (VAI) identifying a visceral adipose dysfunction associated with cardiometabolic risk in a Caucasian Sicilian population. Lipids Health Dis..

[132] Blackburn P., Lemieux I., Lamarche B., Bergeron J., Perron P., Tremblay G., Gaudet D., Despres J.P. (2012). Hypertriglyceridemic waist: A simple clinical phenotype associated with coronary artery disease in women. Metabolism.

[133] Kelley D.E., Mandarino L.J. (2000). Fuel selection in human skeletal muscle in insulin resistance: A reexamination. Diabetes.

[134] Muller M.J., Braun W., Enderle J., Bosy-Westphal A. (2016). Beyond BMI: Conceptual Issues Related to Overweight and Obese Patients. Obes. Facts.

[135] Neeland I.J., Ross R., Despres J.P., Matsuzawa Y., Yamashita S., Shai I., Seidell J., Magni P., Santos R.D., Arsenault B. (2019). Visceral and ectopic fat, atherosclerosis, and cardiometabolic disease: A position statement. Lancet Diabetes Endocrinol..

[136] Amato M.C., Giordano C., Galia M., Criscimanna A., Vitabile S., Midiri M., Galluzzo A., AlkaMeSy Study Group (2010). Visceral Adiposity Index: A reliable indicator of visceral fat function associated with cardiometabolic risk. Diabetes Care.

[137] Amato M.C., Giordano C. (2014). Visceral adiposity index: An indicator of adipose tissue dysfunction. Int. J. Endocrinol..

[138] Lemieux I., Poirier P., Bergeron J., Almeras N., Lamarche B., Cantin B., Dagenais G.R., Despres J.P. (2007). Hypertriglyceridemic waist: A useful screening phenotype in preventive cardiology?. Can. J. Cardiol..

[139] Sam S., Haffner S., Davidson M.H., D’Agostino R.B., Feinstein S., Kondos G., Perez A., Mazzone T. (2009). Hypertriglyceridemic waist phenotype predicts increased visceral fat in subjects with type 2 diabetes. Diabetes Care.

[140] Cunha de Oliveira C., Carneiro Roriz A.K., Eickemberg M., Barreto Medeiros J.M., Barbosa Ramos L. (2014). Hypertriglyceridemic waist phenotype: Association with metabolic disorders and visceral fat in adults. Nutr. Hosp..

[141] LeBlanc S., Coulombe F., Bertrand O.F., Bibeau K., Pibarot P., Marette A., Almeras N., Lemieux I., Despres J.P., Larose E. (2018). Hypertriglyceridemic Waist: A Simple Marker of High-Risk Atherosclerosis Features Associated with Excess Visceral Adiposity/Ectopic Fat. J. Am. Heart Assoc..

[142] Arsenault B.J., Lemieux I., Despres J.P., Wareham N.J., Kastelein J.J., Khaw K.T., Boekholdt S.M. (2010). The hypertriglyceridemic-waist phenotype and the risk of coronary artery disease: Results from the EPIC-Norfolk prospective population study. CMAJ.

[143] Minambres I., Sanchez-Hernandez J., Cuixart G., Sanchez-Pinto A., Sarroca J., Perez A. (2021). Characterization of the hypertriglyceridemic waist phenotype in patients with type 2 diabetes mellitus in Spain: An epidemiological study. Rev. Clin. Esp..

[144] Zheng X., Ren X., Jiang M., Han L. (2022). Association between hypertriglyceridemic-waist phenotype and cardiovascular disease: A cohort study and meta-analysis. Front. Cardiovasc. Med..

[145] Borges L.D., Comini L.O., de Oliveira L.C., Dias H.H., Ferreira E.S., Batistelli C.R.S., da Costa G.D., Moreira T.R., da Silva R.G., Cotta R.M.M. (2021). Hypertriglyceridemic waist phenotype and associated factors in individuals with arterial hypertension and/or diabetes mellitus. J. Nutr. Sci..

[146] Cai R., Zhou J., Bai L., Dong Y., Ding W. (2022). Hypertriglyceridemic-waist phenotype is strongly associated with cardiovascular risk factor clustering in Chinese adolescents. Sci. Rep..

[147] Goodpaster B.H., Sparks L.M. (2017). Metabolic Flexibility in Health and Disease. Cell Metab..

[148] Smith R.L., Soeters M.R., Wust R.C.I., Houtkooper R.H. (2018). Metabolic Flexibility as an Adaptation to Energy Resources and Requirements in Health and Disease. Endocr. Rev..

[149] Galgani J.E., Moro C., Ravussin E. (2008). Metabolic flexibility and insulin resistance. Am. J. Physiol. Endocrinol. Metab..

[150] Yu E.A., Le N.A., Stein A.D. (2021). Measuring Postprandial Metabolic Flexibility to Assess Metabolic Health and Disease. J. Nutr..

[151] Branson R.D., Johannigman J.A. (2004). The measurement of energy expenditure. Nutr. Clin. Pract..

[152] Patel H., Kerndt C.C., Bhardwaj A. (2022). Physiology, Respiratory Quotient.

[153] Sparks L.M., Ukropcova B., Smith J., Pasarica M., Hymel D., Xie H., Bray G.A., Miles J.M., Smith S.R. (2009). Relation of adipose tissue to metabolic flexibility. Diabetes Res. Clin. Pract..

[154] Scheja L., Heeren J. (2019). The endocrine function of adipose tissues in health and cardiometabolic disease. Nat. Rev. Endocrinol..

[155] Okada-Iwabu M., Yamauchi T., Iwabu M., Honma T., Hamagami K., Matsuda K., Yamaguchi M., Tanabe H., Kimura-Someya T., Shirouzu M. (2013). A small-molecule AdipoR agonist for type 2 diabetes and short life in obesity. Nature.

[156] Choi H.M., Doss H.M., Kim K.S. (2020). Multifaceted Physiological Roles of Adiponectin in Inflammation and Diseases. Int. J. Mol. Sci..

[157] Kiernan K., MacIver N.J. (2020). The Role of the Adipokine Leptin in Immune Cell Function in Health and Disease. Front. Immunol..

[158] Zuo H., Shi Z., Yuan B., Dai Y., Wu G., Hussain A. (2013). Association between serum leptin concentrations and insulin resistance: A population-based study from China. PLoS ONE.

[159] Singh S., Lohakare A.C. (2020). Association of Leptin and Carotid Intima-Media Thickness in Overweight and Obese Individuals: A Cross-Sectional Study. J. Assoc. Physicians India.

[160] Castela I., Morais J., Barreiros-Mota I., Silvestre M.P., Marques C., Rodrigues C., Ismael S., Araujo J.R., Angelo-Dias M., Martins C. (2023). Decreased adiponectin/leptin ratio relates to insulin resistance in adults with obesity. Am. J. Physiol. Endocrinol. Metab..

[161] Inoue M., Maehata E., Yano M., Taniyama M., Suzuki S. (2005). Correlation between the adiponectin-leptin ratio and parameters of insulin resistance in patients with type 2 diabetes. Metabolism.

[162] Lee K.W., Shin D. (2020). Prospective Associations of Serum Adiponectin, Leptin, and Leptin-Adiponectin Ratio with Incidence of Metabolic Syndrome: The Korean Genome and Epidemiology Study. Int. J. Environ. Res. Public Health.

